# NFI is an Essential Positive Transcription Factor for Human Papillomavirus Type 16 Early Gene Expression

**DOI:** 10.2174/1874357900701010033

**Published:** 2007-11-22

**Authors:** Amy Baldwin, Melissa K Hypes, Lucia Pirisi, Kim E Creek

**Affiliations:** 1 The Channing Laboratory, Brigham and Women’s Hospital and Department of Medicine, Harvard Medical School, Boston, MA 02115, USA; 2 Virginia Department of Forensic Science, Roanoke, VA 24019, USA; 3 Department of Pathology, Microbiology & Immunology, University of South Carolina School of Medicine, Columbia, SC 29208, and South Carolina Cancer Center, Columbia, SC 29203, USA

## Abstract

Human papillomavirus type 16 (HPV16) is the primary etiologic agent for greater than 50% of all cervical carcinomas. Expression of the HPV16 E6 and E7 oncoproteins is under control of the upstream regulatory region (URR), which contains a myriad of transcription factor binding sites, including 7 half sites for NFI. These NFI binding sites were used as probes in electrophoretic mobility shift assays (EMSAs), and mutational analysis of individual and multiple NFI binding sites was performed in order to demonstrate the relative importance of particular NFI sites to URR activity. By using 5 NFI half sites as an enhancer, we were able to detect a 4-fold increase in URR activity. Our results define the role and relative contribution of NFI binding sites to the basal activity of the HPV16 promoter, and demonstrate that NFI binding sites can act independently to enhance HPV16 URR activity in immortalized keratinocytes.

## INTRODUCTION

Human papillomaviruses (HPVs) are responsible for benign and malignant epithelial lesions such as common warts, genital warts, and cervical cancer [1]. High-risk HPVs are associated with nearly all cervical carcinomas, and HPV Type 16 (HPV16) is by far the most prevalent of the high-risk types [2]. The majority of HPV-associated lesions do not progress to cervical carcinoma, as they are inhibited by multiple mechanisms such as transcriptional repression of the HPV early genes, including the E6 and E7 oncogenes. High-risk HPV E6 and E7 oncoproteins play a significant role in malignant conversion of infected cutaneous epithelial and mucosal cells [3-6]. Since expression of the HPV E6 and E7 oncoproteins is controlled by the upstream regulatory region (URR), transcriptional control of the high-risk HPV URRs (such as HPV16, 18 and 31) has been the focus of numerous studies.

HPV early gene expression is controlled by a complex interaction of cellular and viral factors that bind to the URR, which contains a myriad of transcription factor binding sites, including 7 half sites for nuclear factor I (NFI). NFI, AP-1, Oct-1, Tef-1, Tef-2, and the progesterone / glucocorticoid receptor all bind and act synergistically to promote viral transcription through the HPV URR [7-13].

NFI is a family of site-specific DNA-binding factors that function both in viral DNA replication and in the control of viral and cellular gene expression [14]. We have previously demonstrated that NFI binding sites within the HPV16 URR are critical for TGF-ß modulation of URR activity [15]. While mutational analysis has been performed on several NFI binding sites in the HPV16 URR [16, 17], demonstrating the importance of the NFI transcription factor to HPV16 URR activity, a complete mutational analysis of all 7 NFI sites has not been previously reported.

The main goal of this study was to explore how much each of the 7 NFI binding sites in the HPV16 URR contributes to overall promoter activity. We also sought to determine whether NFI could function as an enhancer of URR activity in the absence of other URR transcription factor binding sites.

## MATERIALS AND METHODS

### Cell Culture and Cell Lines.

Normal human keratinocytes (HKc) were isolated from neonatal foreskins and immortalized by transfection with a plasmid containing a head-to-tail dimer of HPV16 DNA (HKc/HPV16) and cultured in serum-free MCDB153-Luria-Bertani basal medium supplemented with 5 ng of epidermal growth factor/ml, 35 to 50 µg of bovine pituitary extract protein/ml, 0.2 µM hydrocortisone, 0.1 mM calcium chloride, 10 nM triiodothyronine, 10 µg of transferrin/ml, and 5 µg of insulin/ml (complete medium), as described previously [15]. Cells were split 1:10 when confluent, and medium was replaced every 48 h.

### Nuclear Extracts.

HKc/HPV16 were grown to 40 to 50% confluency in 100-mm-diameter tissue culture plates. Nuclear extracts were obtained as previously described [15]. Briefly, cells were rinsed twice with ice-cold phosphate-buffered saline and collected on ice in phosphate-buffered saline containing 1 mM EDTA, scraped and pelleted by centrifugation. Cell pellets were resuspended in ice-cold hypotonic buffer (10 mM HEPES [pH 7.9], 10 mM potassium chloride, 1.5 mM magnesium chloride, 0.5 mM dithiothreitol, 0.2 mM phenylmethylsulfonyl fluoride, 1 µg of leupeptin/ml, 1 µg of pepstatin A/ml), immediately repelleted, resuspended again in ice-cold hypotonic buffer, and allowed to swell on ice for 10 min. Nuclei were obtained by centrifugation (1,500 x *g*, 10 min, 4°C) following disruption of the cells with a Dounce-type mortar and pestle and then resuspended in extraction buffer (250 µl/20 100-mm-diameter tissue culture dishes; 20 mM HEPES [pH 7.9], 0.5 M potassium chloride, 1.5 mM magnesium chloride, 0.2 mM EDTA, 25% glycerol, 0.5 mM dithiothreitol, 0.2 mM phenylmethylsulfonyl fluoride, 1 µg of leupeptin/ml, 1 µg of pepstatin A/ml) by gentle pipetting. Nuclear proteins were extracted by gentle rocking (30 min at 4°C) followed by centrifugation (16,000 x *g*, 30 min, 4°C). The supernatant containing the nuclear extract was dialyzed for 45 min on ice with gentle stirring in 100 ml (400 volumes) of ice-cold dialysis buffer (20 mM HEPES [pH 7.9], 0.1 M potassium chloride, 0.2 mM EDTA, 20% glycerol, 0.5 mM dithiothreitol, 0.2 mM phenylmethylsulfonyl fluoride), and precipitates were removed by centrifugation (16,000 x *g*, 20 min, 4°C).

### Electrophoretic Mobility Shift Assays (EMSAs).

EMSAs were performed as described previously [15]. Double-stranded oligonucleotides representing all 7 NFI half sites, between 20 and 25 bases in length (Fig. 2A), were 5' end labeled and used as probes. HKc/HPV16 nuclear extracts (12 µg of protein) were incubated with 10-fold-concentrated binding buffer (100 mM Tris-HCl [pH 7.5], 0.5 M sodium chloride, 10 mM dithiothreitol, 50% glycerol), 1 µg of poly(dI · dC), 0.5 µg of sonicated herring sperm DNA, and 125-fold unlabeled specific or non-specific oligonucleotides (as competitors to determine binding specificity) in a final volume of 10 µl for 15 min on ice. Probe (100 ng) was added to each reaction mixture and allowed to incubate at room temperature for an additional 15 min. The entire reaction mixture was loaded without dye and resolved on a 5% non-denaturing Tris-glycine polyacrylamide gel (2.5 h at 150 V). Gels were dried and visualized using a Bio-Rad K-screen and phosphorimager.

### Plasmid Constructs and Mutagenesis.

**Fig. (1):**
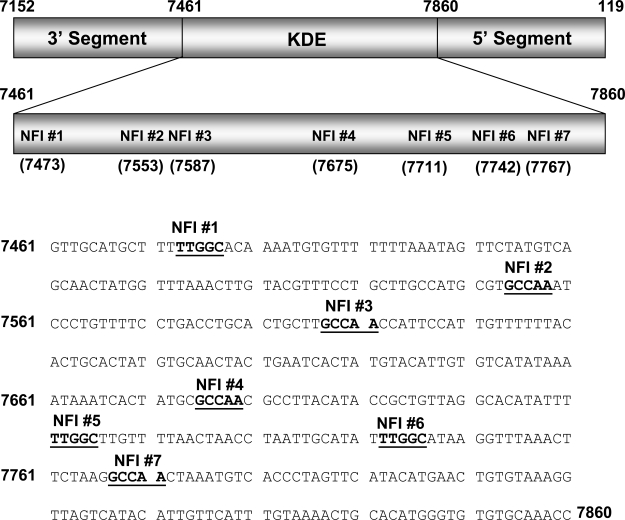
NFI binding Sites of the HPV16 URR. The central keratinocyte dependent enhancer (KDE), and the 3’ and 5’ segments of the HPV16 URR are shown. The location of each NFI half site within the KDE is noted. The sequence for the HPV16 URR KDE is given, with the 7 NFI binding sites bolded and underlined.

A luciferase reporter vector under control of the HPV16 URR (pGL3/URR) was constructed by cloning the entire URR (Fig. **1**) into the HindIII multiple cloning site of pGL3-basic (Promega), as described previously [15]. For NFI mutational analysis, mutant constructs were created using the Quick Change site-directed mutagenesis kit (Stratagene) by introducing point mutations in single and multiple NFI site(s) (GCCAA changed to GCAGA which is unable to bind NFI). For NFI enhancer analysis, enhancer elements containing 5 consensus or mutant NFI half sites, each separated by 10 random nucleotides (Fig. **4**), were synthesized. These elements were cloned upstream of the CMV promoter / luciferase gene (pGL3 promoter, Promega). All constructs used were verified by sequencing.

### Transfections and Luciferase Assay.

Transfections and luciferase assays have been described previously [15]. Plasmid constructs were transfected, in triplicate, into HKc/HPV16 cultured in 60-mm-diameter dishes at 30 to 40% confluency by using Transfast (Promega). To control for variations in transfection efficiencies (which ranged from 10 to 30%), the triplicate plates were trypsinized, and the cells were pooled and replated onto six 60-mm-diameter dishes 12 to 15 h posttransfection. Luciferase activity was determined 68 to 72 h posttransfection with the luciferase assay system (Promega). Experiments were performed at least three times, and each construct was tested in triplicate dishes. Relative light units were determined using a luminometer (Berthold Lumat LB9501). Background luciferase activity was determined by transfection with pGL3-basic alone.

## RESULTS AND DISCUSSION

### Binding to the NFI Sites of the HPV16 URR.

**Fig. (2):**
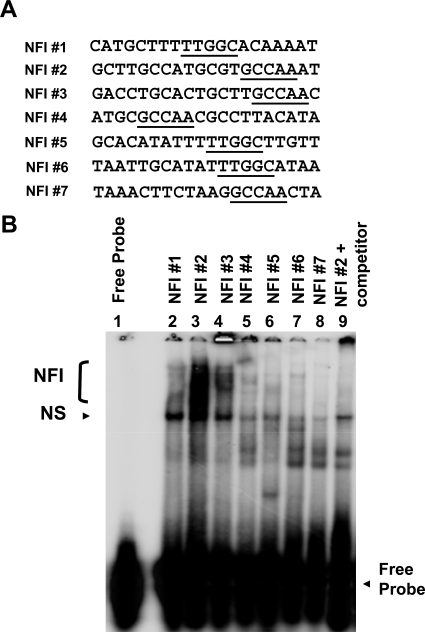
Binding to NFI Sites of the HPV16 URR. (**A**) Double stranded oligonuclotides representing the 7 NFI half sites of the HPV16 URR were labeled as probes and used in electrophoretic mobility shift assays (EMSAs). The nucleotide sequence for each probe is shown. (**B**) Nuclear extract (12 µg of protein) from HKc/HPV16 was incubated with each probe. Protein-probe complexes were separated from the free probe on a 5% non-denaturing polyacrylamide gel. Specific NFI binding as well as non-specific (NS) binding is noted. Addition of cold competitor (125X) illustrates specific NFI binding (lane 9).

To investigate the binding of cellular factors to the NFI sites of the HPV16 URR (schematic shown in Fig. **1**), oligonucleotides representing all 7 NFI half sites were constructed (Fig. **2A**) and used as probes in electrophoretic mobility shift assays (EMSAs) (Fig. **2B**). Nuclear extract from HPV16-immortalized human keratinocytes (HKc/HPV16) was obtained and incubated with each probe. Cold competitor probe was added to determine specific NFI binding (lane 9, Fig. **2B**), and free probe was separated from probe·protein complexes (Fig. **2B**). The presence and the location of NFI binding were previously verified and reported using NFI sites #2 and #3 in supershift analysis using NFI antiserum (provided by Dr. Naoko Tanese) [15].

Binding to the NFI sites of the HPV16 URR varied in pattern and intensity (Fig. **2B**). A smear of NFI binding can be seen for NFI sites #2 and #3, and to a lesser extent for site #1 (Fig. **2B** lanes 2, 3 and 4). Very little binding, however, was observed for NFI site #7 (Fig. **2B** lane 8). NFI sites #4, #5 and #6 showed a reduced, varied pattern of binding (Fig. **2B** lanes 5-7), which is likely due to the close proximity of multiple transcription factor binding sites. For example, Tef-1 binding sites are adjacent to NFI sites #4 and #6, and therefore may include Tef-1 in the protein complex. These results demonstrate that binding to all NFI sites of the HPV16 URR is not equal, suggesting that the relative contribution of each NFI site to early gene expression may differ.

### Mutational Analysis of HPV16 URR NFI Binding Sites.

**Fig. (3):**
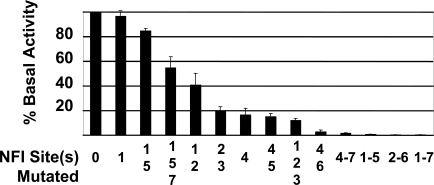
Effects of Single and Multiple NFI Mutations on Basal Activity of the HPV16 URR. The entire HPV16 URR (Fig. 1) was cloned into pGL3 (luciferase reporter vector, Promega) (pGL3/URR) where various NFI sites were mutated from GCCAA to GCAGA, which is unable to bind NFI. These constructs were transfected into HKc/HPV16, and luciferase activity was determined 68 to 72 h post transfection. Basal activity was calculated as the percent of wild type (up to 9.3 x 10^5^ RLU), and is shown for each mutant construct. Error bars indicate standard deviation (SD).

A mutational analysis of the NFI sites was performed to determine the role of the 7 NFI sites in transcription of the HPV16 early genes. Point mutations of single and multiple NFI sites were introduced within the context of the entire HPV16 URR, cloned upstream of a luciferase reporter gene. Mutation of NFI sites #1 and #5 had very little effect on basal activity of the HPV16 URR, while constructs containing mutations to either NFI site #4 or NFI sites #2 and #3 reduced the basal activity of the promoter to less than 20% (Fig. **3**). All constructs containing mutations to at least 5 NFI binding sites retained less than 1% of basal activity (Fig. **3**). Although these 3 constructs lost over 99% of basal activity, measured RLU values were still 3 to 5-fold greater than those obtained from transfection with pGL3-basic alone.

Previous mutational analysis of NFI binding sites of the HPV16 URR involved cloning 91 bp fragments of the HPV16 URR containing NFI sites #2 and #3, or NFI sites #4 and #5 upstream of the TK promoter / CAT reporter and transfection into HeLa cells [17]. This analysis revealed that these 4 NFI sites were important for enhancer activity, to varying degrees. Mutation of NFI site #4 reduced relative activity to 15.8%, which mirrored our results for the NFI site #4 mutant (Fig. **3**). However, our mutational analysis of NFI sites #2, #3, and #5 did not concur with this previous study. This may be due to the fact that our mutational analysis was done in the context of the entire HPV16 URR without the use of additional promoters, or that the previous analysis was carried out in HeLa cells, while ours were performed in HPV16-immortalized keratinocytes.

The relative amount of NFI binding observed for each site correlated somewhat to the importance of that particular binding site to basal activity of the promoter. For example, NFI sites #2 and #3 showed a relatively large amount of binding (Fig. **2B** lanes 3 and 4), and upon mutation of those NFI sites, basal activity of the promoter was reduced by 81% (Fig. **3**). Conversely, when NFI sites were mutated that showed a lesser amount of binding (sites #1 and #5, Fig. **2B** lanes 2 and 6), the basal activity of the promoter was reduced by only 15% (Fig. **3**). An exception to this trend was NFI site #4. This site demonstrated a relative reduced level of binding (Fig. **2B** lane 5); however, the basal activity of the promoter was reduced by 83% upon mutational analysis (Fig. **3**). Collectively, these data reveal NFI to be a crucial transcription factor for HPV16 URR basal activity. The contribution of each NFI binding site, however, is not equal.

### NFI Binding Sites as an Enhancer.

**Fig. (4):**
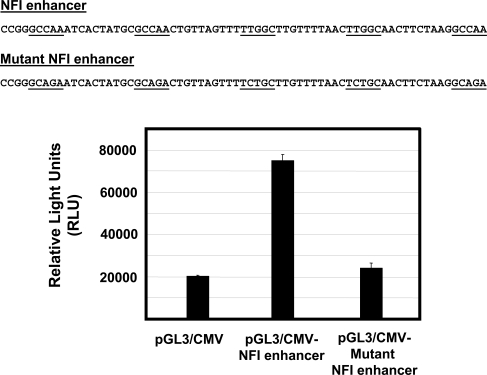
NFI Binding Sites Exhibit Enhancer Function. A 69 bp enhancer element containing either 5 NFI consensus or mutant half sites was synthesized and cloned upstream of a CMV promoter / luciferase gene (pGL3 promoter, Promega). NFI sites were mutated from GCCAA to GCAGA, which is unable to bind NFI. These constructs were transfected into HKc/HPV16, and luciferase activity was determined 70 h post transfection. The sequence for each enhancer element is shown, and the NFI consensus or mutant sites are underlined. Luciferase expression (RLU) is given for each construct. Error bars indicate standard deviation (SD).

It was previously reported that a cell-type-dependent regulatory element (CTRE), a 178 bp element containing NFI sites #1 to #3 (nt 7454 to 7632), is necessary for full enhancer activity in an HPV-immortalized keratinocyte cell line [16]. To determine whether NFI could act as an enhancer in the absence of HPV16 URR sequences, we synthesized enhancer elements containing 5 consensus or mutant NFI half sites, each separated by 10 random nucleotides (Fig. **4**). These elements were cloned upstream of the CMV promoter / luciferase gene (pGL3 promoter, Promega), the resulting constructs were transfected into HKc/HPV16, and luciferase activity was measured 70 h post transfection. The NFI enhancer produced a 4-fold increase in luciferase activity (Fig. **4**), compared to the CMV promoter alone. This increase was not observed for the mutant NFI enhancer element (Fig. **4**), demonstrating that NFI enhancer function does not necessarily depend on other sequence elements located in the HPV16 URR.

The possibility that NFI requires a co-activator(s) for expression in epithelial cells has been previously proposed [17]. Exogenous NFIC was found to activate HPV16 enhancer fragments in NFI deficient SL-2 (insect) cells. However, this activation did not take place in fibroblasts, implying that a necessary co-activator was absent in fibroblasts [17]. Our data suggest that if a co-activator is necessary for enhancer function, it does not require elements within the URR for binding. Additionally, Oct-1 has been shown to activate epithelial-specific enhancer activity of HPV16 by interaction with NFI site #6 at a conserved, composite element in HeLa cells, dependent upon both the binding site for NFI and an adjacent site able to bind Oct-1 [18]. While it is not our intention to rule out the influence of positive co-activators, our results do imply that other URR binding sites are not necessary for anchorage or stabilization of NFI co-activator(s), and that the stereospecificity contributed by adjacent transcription factor binding elements in the HPV16 URR must not be crucial for the enhancer function of NFI. The apparent contradiction between our study and previous studies [17, 18] may be due to cell-type specific co-activator(s) and / or the physiological state of the cell. Interestingly, in light of our binding and mutational analysis, negative regulatory elements located near NFI binding sites in the HPV16 URR likely contribute to reduced NFI binding (Figs. **2** and **3**) as binding to NFI sites #1 and #5 is minimal (Fig. **2B**, lanes 2 and 6), and mutation of those 2 sites produces only a small decrease in basal activity of the HPV16 URR.

This work supports previous reports that NFI is an important positive transcription factor for the activity of the HPV16 URR [7, 11, 16-19]. This is the first study however, to demonstrate the role and relative contribution of each NFI site to HPV16 early gene expression. This mutational analysis, conducted in the context of the entire HPV16 URR, is strongly supported by our NFI binding data. We also demonstrate here that NFI binding sites do comprise strong enhancer function in HPV16-immortalized human keratinocytes, in the absence of other URR elements. Collectively, these results demonstrate that NFI is an essential positive transcription factor for HPV16 URR activity.
